# Autophagy-Mediated Exosomes as Immunomodulators of Natural Killer Cells in Pancreatic Cancer Microenvironment

**DOI:** 10.3389/fonc.2020.622956

**Published:** 2021-02-19

**Authors:** Daniela L. Papademetrio, María Noé Garcia, Daniel Grasso, Élida Alvarez

**Affiliations:** ^1^ Departamento de Microbiología, Inmunología, Biotecnología y Genética, Facultad de Farmacia y Bioquímica, Universidad de Buenos Aires, Buenos Aires, Argentina; ^2^ CONICET, Instituto de Estudios de la Inmunidad Humoral (IDEHU), Universidad de Buenos Aires, Buenos Aires, Argentina; ^3^ Departamento de Ciencias Biológicas, Facultad de Farmacia y Bioquímica, Universidad de Buenos Aires, Buenos Aires, Argentina

**Keywords:** pancreatic cancer, autophagy, exosomes, natural killer cells, tumor microenvironment

## Abstract

Pancreas ductal adenocarcinoma is a highly aggressive cancer with an incredible poor lifespan. Different chemotherapeutic agents’ schemes have been tested along the years without significant success. Furthermore, immunotherapy also fails to cope with the disease, even in combination with other standard approaches. Autophagy stands out as a chemoresistance mechanism and is also becoming relevant as responsible for the inefficacy of immunotherapy. In this complex scenario, exosomes have emerged as a new key player in tumor environment. Exosomes act as messengers among tumor cells, including tumor microenvironment immune cells. For instance, tumor-derived exosomes are capable of generating a tolerogenic microenvironment, which in turns conditions the immune system behavior. But also, immune cells-derived exosomes, under non-tolerogenic conditions, induce tumor suppression, although they are able to promote chemoresistance. In that way, NK cells are well known key regulators of carcinogenesis and the inhibition of their function is detrimental for tumor suppression. Additionally, increasing evidence suggests a crosstalk between exosome biogenesis and the autophagy pathway. This mini review has the intention to summarize the available data in the complex relationships between the autophagy pathway and the broad spectrum of exosomes subpopulations in pancreatic cancer, with focus on the NK cells response.

## Introduction

Pancreatic ductal adenocarcinoma (PDAC) is an aggressive tumor with a survival of 4-6 months after diagnosis (1, 2). Besides the lack of early diagnosis, the rapid development of chemoresistance makes PDAC one of the highest deadly cancers (3). PDAC cells show high levels of basal autophagy (4–6) and an immunosuppressive tumor microenvironment (TME) conditioned by the presence of immunosuppressive cells, i.e., regulatory T lymphocytes (Tregs), tumor-associated macrophages, and myeloid-derived suppressor cells (MDSCs) widely present in the early stages of the disease (7, 8). Immunotherapy against CTLA4 and PD-L1, with remarkable results on several solid tumors (9, 10), was unsuccessful in PDAC patients (11), even in combination with other standard approaches (12).

Macroautophagy, hereafter named simply as autophagy, is a catabolic process with the capacity to degrades cellular constituents including whole organelles (13, 14). Briefly, upon autophagy induction the serin kinase activity of ULK1 complex induces a successive recruitment of autophagy proteins to discrete areas of ER membrane. This includes a complex with phosphatidyl-inositol 3-kinase activity (PI3K), composed of Beclin-1, ATG14, Vps150 and Vps34, which in turn deposit phosphatidyl-inositol 3-phosphate (PI3P) which is recognized by further autophagic proteins (13, 14). These events let to evolution of the isolation membrane followed by WIPI1/2-mediated recruitment of ATG5-12-16L complex, needed for the incorporation of the lipidated form of LC3B to the budding membrane. Isolation membrane invaginates over the cargo in a LC3-decorated double membrane vesicle denominated the autophagosome. Eventually, the autophagosome fuses with a lysosome where cargo is degraded (13, 14). Literature is contradictory about the role of autophagy in cancer and then it is accepted to be a context dependent factor (6, 15). *In vitro*, gemcitabine which is the standard chemotherapeutic agent against PDAC increases the autophagic flux in PDAC cells in order to avoid its deleterious effects (4). Then, autophagy inhibition increases the sensitivity of PDAC cells to gemcitabine, but also to other treatment such as inhibitors of the NF-kB and MAPK pathways (5).

Extracellular vesicles (EVs) can be classified by size as small (sEVs - <200 nm) or medium/large (m/lEVs - >200 nm), but they can also be discriminated by density, membrane markers and cell type origin (16). From that plethora of different types of EVs, exosomes are those sEVs with an endosomal origin and a size ranging from 30 to 200 nm (17). The biogenesis of these vesicle is produced by inward budding of the membrane of late endosomes/multivesicular bodies (MVBs) (18), These particles are secreted by most cells, including tumoral cells, and can exert an effector response in distant tissues (19, 20). Exosomes transfer their content specifically to target cells, through mechanisms including ligand/receptor recognition, direct fusion with the recipient cells, phagocytosis, endocytosis (21–24). Moreover, they can transfer receptors from the plasma membrane (25) and deliver, to target cells, proteins (26), mRNAs, miRNAs (27, 28) and reporters genes (29). The mechanism that controls the inclusion of specific molecules within the exosomes remains to be clarified, and in addition, a cell can secrete diverse exosome populations each one with a unique content. The so-called tumor-derived exosomes or TEXs often bear tumor associated antigens and in some cases they can activate and stimulate immune cells (30, 31). However, the effect of TEXs over the immune system is not always activation (32). Hence, TEXs can induce apoptosis of effector T cells (33–37), inhibit the cytotoxic activity of natural killer (NK) cells (38–40), activate immunosuppressive functions in myeloid cells (24, 41, 42), impair differentiation of dendritic cells (43), and induce the response of Tregs (44, 45).

NK cells are a class of innate lymphocytes cells with the ability to rapidly eliminate infected and tumor cells. There are two main subclasses of NK cells, phenotypically and functionally different. Phenotypically they can be classified according to the level of CD56 and CD16 expression and functionally according to their cytotoxic potential (46). The CD56^dim^CD16^bright^ NK cells subset is highly cytotoxic and expresses high levels of perforin and granzyme B. This subpopulation does not migrate to secondary lymphoid organs, but they express chemokine receptors that allow them to migrate to inflamed tissues, can mediate ADCC processes, and have low cytokine secretion capacity. By contrast, CD56^bright^CD16^dim^ NK cells are the cytokine secreting subset, do not migrate to inflamed tissues and their cytotoxic capacity is limited (46). Opposite to LT CD8^+^, mature NK cells do not need previous activation to exert their functions. Moreover, NK functionality is independent of the presence of non-self-antigens presented by MHC molecules to CD8+ lymphocytes. Hence, NK cells can eliminate target cells without previous sensitization. Nonetheless, it is now well-known that previous activation enhances NK cell activity by regulating the expression of cytotoxic mediators, as well as several receptors (47). Furthermore, previous exposure to haptens, viral infection (HCMV) or cytokines (IL12, IL15, and IL18) generates adaptive NK cells with immunological memory (47).Nevertheless, the potent immunosuppressive TME in PDAC impairs NK function and cytotoxicity by different ways such as downregulation of effector molecules and activation receptor (48). Altogether, NK cells are serious candidates to develop therapeutic strategies to eliminate tumors that are invisible for T cells.

The last few years have seen little or no progress in the development of more effective treatments for patients with PDAC. In this review, we aim to analyze the complex relationships between autophagy and the broad spectrum of exosomes in TME of PDAC, with focus on NK cell response.

## Pancreatic Cancer Cells Are Modulated by TME-Derived Exosomes

There is a complex and dynamic relationship among tumor autophagy, immune response and TME. TME is a complex system that is affected by several factors including hypoxia, acidosis, and immune and inflammatory responses. Moreover, TME influences cell adhesion, invasion, angiogenesis, and even tumor autophagy which in turn can promote tumor growth and enhance metastasis. TME is responsible for release of the chemoattractant factors that recruit the immune effector cells. The response of tumor autophagy to the inflammatory components is unpredictable and the generation of a pro-inflammatory environment may not always be effective against the tumor. For example, IL-1 can inhibit the cyclooxygenase 1 (COX-1) signaling pathway, and phosphorylation of the κB inhibitor (IκB), promoting tumor development and metastasis. In contrast, inhibition of IL-1 expression in tumor cells induces overexpression of p21 and p53, leading to tumor suppression (49). At the pancreatic level, IL-1β induces autophagy in acinar pancreatic cells (50). In the case of pancreatic tumors, they are in hypoxic TME which induces autophagy. In this setting, tumors were reported to increase their autophagy levels in order to selectively degrade granzyme B released by NK cells, thereby inhibiting one of the cytotoxic mechanisms of NK cells (51, 52).

One important member of TME are the cancer-associated fibroblasts (CAFs) which foster proliferation (53) and chemoresistance (54) in PDAC. CAFs are innately insensitive to gemcitabine and a key player in the development of chemoresistance in tumor cells. Exosomes released by gemcitabine-treated CAFs increase proliferation and survival of PDAC cell lines by carrying the chemoresistance-inducing factors, Snail, and miR-146a, which in turn also induce its own expression in the recipient cells (54). Moreover, CAFs-derived exosomes contain the miR-106b which promote gemcitabine resistance in PDCA cells by targeting TP53INP1 (55).

Recently, the exosomes from bone marrow mesenchymal stem cell (BMSC), residents of the TME, raised attention in PDAC. The over-expression of miR-126-3p in BMSC-derived exosomes not only inhibits the proliferation, invasion and metastasis of PDAC cells, but also promotes apoptosis *in vitro* and *in vivo* by down-regulation of disintegrin and metalloproteinase-9, ADAM9 (56). Furthermore, the amount of miR-1231 in those sEVs was significantly correlated with the TNM stage of PDAC in the clinic. The proliferation, migration, invasion, and adhesion to the matrix of PDAC cells were negatively regulated by BMSC-derived exosomes transfected with miR-1231 oligonucleotides. Then, the exosomes extracted from BMSCs, with high levels of miR-1231, inhibit the proliferation of pancreatic cancer cells and induce cell cycle arrest (57). Finally, similar results can be observed in exosomes from the tumor-associated stroma (TAS) cells, which are enriched in miR-145 and possess tumor suppressive properties by inducing apoptosis of PDAC cells (58).

The studies carried out suggest that the ability of exosomes to induce or suppress the proliferation, invasion, metastasis and/or chemoresistance of pancreatic cancer cells, depend on the cell type where those vesicles come from. Different sources of exosomes have different effects on pancreatic cancer cells activity, or even the opposite, which needs further clarification and in-depth study.

## Relationship Between Autophagy and Exosomes Biogenesis

Alternative to the direct fusion with lysosomes (see *Introduction*), autophagosomes can previously fuse with some endocytic compartments such as early and late endosomes, and the MVBs. These merged structures, called amphisomes, eventually fuses with lysosomes where sequestered material is finally degraded (59). Therefore, autophagy induction has been shown to cause recycling of the MVBs, which, instead of fusing with the plasma membrane, enter the autophagic pathway. Furthermore, it was observed in some cell lines a relationship between exosomes release and the induction level of the autophagy pathway (60–63). Consequently, it is not surprising to find evidence of autophagic pathway cross-linking with exosome biogenesis (64). Starvation-induced autophagy reduces the release of exosomes in K562 cells (63). Starvation could increase in the fusion between MVBs and autophagosomes, thus directing the MVBs toward the degradative pathway. Similarly, inhibition of PIKfyve kinase, essential for endolysosomal vesicular trafficking, increases exosome release and reduces the degradative process *via* autophagy, probably due to reduced fusion of lysosomes with MVBs and autophagosomes (65). In line with this, the lysosomal dysfunction, provoked by ammonium chloride or bafilomycin A1, increases sEVs secretion of SH-SY5Y cells (66). Nevertheless, we cannot discard that those results are due to vesicular trafficking interference.

There is data supporting that at least part of the autophagy machinery contributes to the biogenesis of exosomes, in a process where completion of the autophagic process itself seems to be dispensable (67, 68). In non-autophagic functions, ATG5 and ATG16L1 proteins have been associated with the biogenesis of exosomes (67). ATG5 participates in the dissociation of the vacuolar proton pump (V1V0-ATPase) from the MVBs preventing its acidification, and this is believed to allow the fusion with the plasma membrane and consequent exosomes releasing. Accordingly, depletion of ATG5 or ATG16L1 significantly reduces exosome release and attenuates exosomal enrichment in LC3B-II. Moreover, lysosomal or V-ATPase inhibitors rescue the release of exosomes in ATG5 depleted cells further supporting the role of luminal pH to define the fate of MVBs. It is interesting to note that while ATG5 decreases the acidification of the MVBs, it increases the acidification in those LC3 positive intracellular compartments, such as autolysosome, phagosomes associated with LC3 and endosomes, all of them destined for degradation. A proposed model indicates that in MVBs, LC3 can remove ATP6V1E1 from intraluminal vesicles/exosomes and decrease acidification, while it recruits ATP6V1E1 or stabilize V1V0ATPase in the aforementioned degradative vesicles to promote acidification (67). The complex of two other autophagy proteins, ATG12 and ATG3, interacts with ALIX and ESCRT-associated proteins, crucial in exosomes biogenesis (68). Hence, loss of ATG12-ATG3 alters the morphology of MVBs, impedes late endosome trafficking, and reduces exosome biogenesis. Worth note that decreased ALIX expression reduces basal autophagic flux, demonstrating reciprocal regulation between both pathways. Interestingly, the lack of ALIX or the ATG12-ATG3 complex impairment do not affect starvation-induced autophagy, suggesting different regulatory machinery for basal and stress-induced autophagy, as well as the interaction of these pathways with endocytic compartments (68).

Highly desmoplastic and poor vascularized, PDAC stroma imposes a hypoxic condition to most cancer cells into pancreatic tissue. It was described that hypoxia, an autophagy inductor, promotes the release of EVs in several PDAC cell lines. The effect seems to be quite specific since a significant increase of sEVs, without or minimal release of mEVs and lEVs, is observed. Moreover, changes in size distribution among the sEVs is observed with a shift toward smaller average size under extreme hypoxia (69). Furthermore, the GAIP C-terminal interaction protein (GIPC) acts as a scaffold to control receptor-mediated trafficking (70–72). After receptor internalization, GIPC is transiently associated with the pool of endocytic vesicles that are close to the plasma membrane (73). A regulatory role of GIPC on autophagy, *via* the glucose-dependent metabolic pathway, and on biogenesis and release of exosomes has been described in AsPC-1 and PANC-1 pancreatic tumor cells (74). GIPC depletion in these cell lines generates metabolic stress with autophagy induction and increased exosome release. Lack of GIPC increases LC3-II expression and biogenesis of autophagosomes and at the same time leads to increased secretion of exosomes by the PDAC cells. Mechanistically, the absence of GIPC increases exosomes released by higher expression levels of ALIX, TSG101 and CHMP4B. Noteworthy is that exosomes from GIPC-depleted cells lack the drug resistance associated molecule ABCG2, suggesting that this molecule might be a sEVs cargo (74).

Altogether, several molecules that belong from the autophagy pathway seem to play important roles in exosomes biogenesis. However, we still have a long way to precisely define how deeply the autophagy and exosomes biogenesis are crisscrossed. In addition, the stroma profile, for instance through its hypoxic status, let us glimpse that influences of TME over cancer cells in PDAC could be even far more complex that speculated some time ago.

## PDAC-Derived Exosomes Influence Tumor Behavior

Among the cells with different grades of malignancy that compose the PDAC, highly invasive cell-derived exosomes promote the migration and invasion of weakly invasive cells (75). ZIP4, a zinc transporter, is the most up-regulated exosomal protein and promotes the growth of recipient cells (76). Moreover, exosomes derived from highly invasive cells are rich in miR-125b-5p which promotes migration and invasion and is associated with metastasis in PDAC through MEK2/ERK2 signaling (77). Likewise, the miR-5703 present in exosomes from primary cultures of pancreatic stellate cells is capable of fostering proliferation of PDAC cells by activation of the PI3K/Akt pathway. This has been shown to be due to miR-5703 downregulated CMTM4 where CMTM4 suppresses the PI3K/Akt pathway (78). In a different aspect of tumor biology, TEXs can also transfer chemoresistance by a paracrine action. Gemcitabine, being one of the most commonly used chemotherapeutic agents in pancreatic cancer (79), upregulates miR-155 expression in PDAC cells which is transferred, through exosomes, to the neighboring cells. This microRNA confers chemoresistance to receptor cells by upregulation of SOD2 and CAT, involved in ROS detoxification, and downregulation of DCK, a gene related to gemcitabine metabolism (80). Additionally, miR-155 induces the biogenesis and secretion of exosomes leading to a positive feedback loop of drug resistance (80, 81).

The composition, biogenesis and secretion of exosomes are finely regulated processes, influenced by changes in the TME. In this context, exosome secretion is an efficient adaptive mechanism by which cells modulate intracellular stress situations and modify their microenvironment (82). PDAC cells are usually exposed to hypoxia, which is an important autophagy inductor, as commented above. This situation induces adaptation mechanisms that promote endothelial activation, angiogenesis, proliferation, and cell survival (83). Noteworthy, concomitantly to autophagy flux induction, cells under hypoxic or anoxic stress increase the secretion of exosomes rich in CD63, CD9, and miR-210 in breast cancer cells (84). Furthermore, in this situation, secreted exosomes contain proteins associated with cell migration, degradation of the extracellular matrix, growth signaling molecules, clathrin-mediated endocytosis, and molecules of the endothelial and vascular growth factor signaling pathway (85, 86). Acid conditions are common in the tumor microenvironment. This factor also modulates the release, charge, function, and trafficking of exosomes released by the tumor cell. An acidified microenvironment increases the release of exosomes, but with a different lipid composition. These exosomes are enriched in sphingomyelin and GM3 ganglioside, thus increasing their rigidity and fusion efficiency with the target cell (87). It has been shown that situations such as oxidative and thermal stress can increase the release of immunosuppressive exosomes from leukemia cells and T and B lymphomas (32). Besides, the effects of pancreatic cancer-derived sEVs on T lymphocytes are far from be elucidated. The promotion of Treg expansion and impairment of T lymphocytes cytotoxicity against PDAC cells by pancreatic cancer-derived sEVs was recently described. In these lymphocytes, the up-regulation of FOXP3 and the consequent Treg promotion was mediated by the ATM-AMPK-SIRT1/2/6- FOXO1A/FOXO3A axis, suggesting an induction of autophagy pathway by PDAC TEXs (88).

In the TME, the exosomes from hypoxic PDAC cells are capable of activating the PTEN/PI3K pathway, inducing the shifting of macrophages toward the M2 phenotype. This process is dependent on HIF1a or HIF2a, and accelerates invasion, migration and epithelial-mesenchymal transition (EMT) of PDAC cells (89). Moreover, CD151−/tetraspanin 8 containing exosomes support the EMT of non-metastatic PDAC cells for a motile phenotype (90). Furthermore, exosomes bearing VEGF and TGF-β promote angiogenesis enhancing the invasiveness and the establishment of a metastatic TME. PDAC also releases TEXs bearing c-Met (proto-oncogene mesenchymal-epithelial transition factor) and PD-L1 (programmed cell death 1 ligand 1) (91). The tyrosine kinase receptor c-Met controls key signaling cascades including MAPK, STAT, NF-κB and PI3K/Akt pathways, which overall provide proliferation, migration and an anti-apoptotic status of tumor cells (92). On the other hand, PD-L1 is a ligand of the PD-1 receptor which prevents from excessive immune response and guarantees the tolerance of harmless antigens and self-tissues. Tumor cells take advantage of this mechanism by expressing PD-L1 in order to evade immune control (93). In this context, TEXs from PDAC bear both c-Met and PD-L1 on their surface enhancing the carcinogenesis. Importantly, detection of c-Met and PD-L1 may have diagnostic or prognostic relevance when are detected jointly with the marker CA 19-9 used in PDAC (91).

Finally, PDAC-derived TEXs composition results from activation of several survival pathways which confers aggressiveness, chemoresistance and even immune evasion to neighboring tumor cells.

## Natural Killer Cells in PDAC Microenvironment

NK cells are central in the immunological fight against tumor and infected cells. Although NK cells are expected to play an important role in the immune surveillance against tumors, suppressive components in the TME dampen their efficacy. Several studies proposed Tregs as the responsible in suppressing tumor-infiltrating NK cells (94, 95). However, TEXs have gained attention as key players for immunosuppression in the TME. The exposure of phosphatidylserine (PS) is perhaps the most representative “eat-me” signal which is recognized by opsonins and other serum proteins for removal of apoptotic bodies by phagocytic cell. Physiologically, the externalized PS functions as a dominant immunosuppressive signal, promoting tolerance and preventing local and systemic activation of immune system. Pathologically, the innate immunosuppressive effect of externalized PS has been commandeered by numerous microorganisms to facilitate infection, and in some cases to establish infection latency. In TME, PS is also profoundly dysregulated and inhibits the development of tumor immunity. The exposure of PS is favored by the hypoxic stress, but also PS is exposed in TEXs where it binds to PS-receptors (e.g., TIM-receptors on immune cells), triggering the immune-suppressive signals (i.e. enhanced TGF-β and IL-10 secretion) and leading to an impaired immune activation (96).

NK cells exert their cytotoxic function directly by contact with tumor cells, but also through the action of their own secreted exosomes. Interestingly, the exosomes released by the NK cells seem to be independent of its activation state. Activated and resting NK cells release almost the same number of exosomes, which contain typical protein markers of NK cells such as FasL and perforin. Moreover, these exosomes exert cytotoxic activity against several tumor cell lines *in vitro* (97). In this sense, NK-derived exosomes can regulate tumor cells suppression by two mechanisms: Fas-FasL interaction between exosomes and tumoral cells membrane, and cytotoxicity triggered by excessive uptake of exosomes in the target cells (98). Recently, the presence of miR-3607-3p in EVs was associated with suppression of pancreatic cancer (99). EVs derived from NK cells, enriched in miR-3607-3p, could suppress PDAC development and malignant transformation. The amount of miR-3607-3p in NK cells and its EVs is higher than in PDAC cells, but this miRNA increases significantly in these last when they are cultured in presence of NK-derived EVs (99). IL-26 is a member of the IL-10 cytokine family with unknown function in human tumors. Compared to healthy tissue IL-26 in highly expressed PDAC, miR-3607-3p directly suppresses its expression in these tumoral cells. In sum, there is a significant negative correlation between the expression levels of miR-3607-3p and IL-26 in pancreatic cancer tissues. However, in gastric cancer cells it was reported that over-expression of IL-26 facilitates proliferation and survival by regulation of STAT1/STAT3 signaling (100). Worth to note, that characteristics of cytokine composition in the surroundings where NK cells are activated determine the fate of those immune cells. For instance, survival of NK cells is promoted in presence of IL-15, an innate cytokine, or IL-2, an adaptive cytokine. However, NK cells activated in presence of IL-2 die by apoptosis after contact with vascular endothelium, a key step for their extravasation (101). Further work will elucidate whether the exosomes present in the TME could be mediators of this phenomenon.

NK cells represent a significant attempt of the immune system to fight against PDAC. Nevertheless, cancerous cells, through the PDAC-derived TEXs, can inhibit the functionality of NK cells. In response, NK cells release exosomes which contain FasL and perforin and seem to exert cytotoxic activity against tumor cells. More evidence is needed to completely understand the role of NK-derived exosomes over the PDAC as a whole and vice versa.

Finally, those data could give us the basis to design strategies where this game of different intratumorally exosome populations are exploited for the well-being of patients.

## Conclusions and Perspectives

Indeed, the development of specific immunotherapy protocols based on NK cells to treat cancer has been dampened by the complexity of the mechanisms that regulate NK cell function and elimination of target cells. Luckily, times are changing and, at present, in the era of cancer heterogeneity and immunotherapy, NK cells are emerging as the golden effectors to eliminate non-antigenic tumor cell clones. A perfect duet in the symphony of destruction, LTc and NK cells destroy immune “visible” and “invisible” cancer cells to overcome immunogenic tumor heterogeneity. A better understanding of autophagy and exosome pathways and their interrelationships seems to be key for controling these events, where we could find the way of successfully using the immune system against the deadly PDAC.

**Figure 1:**
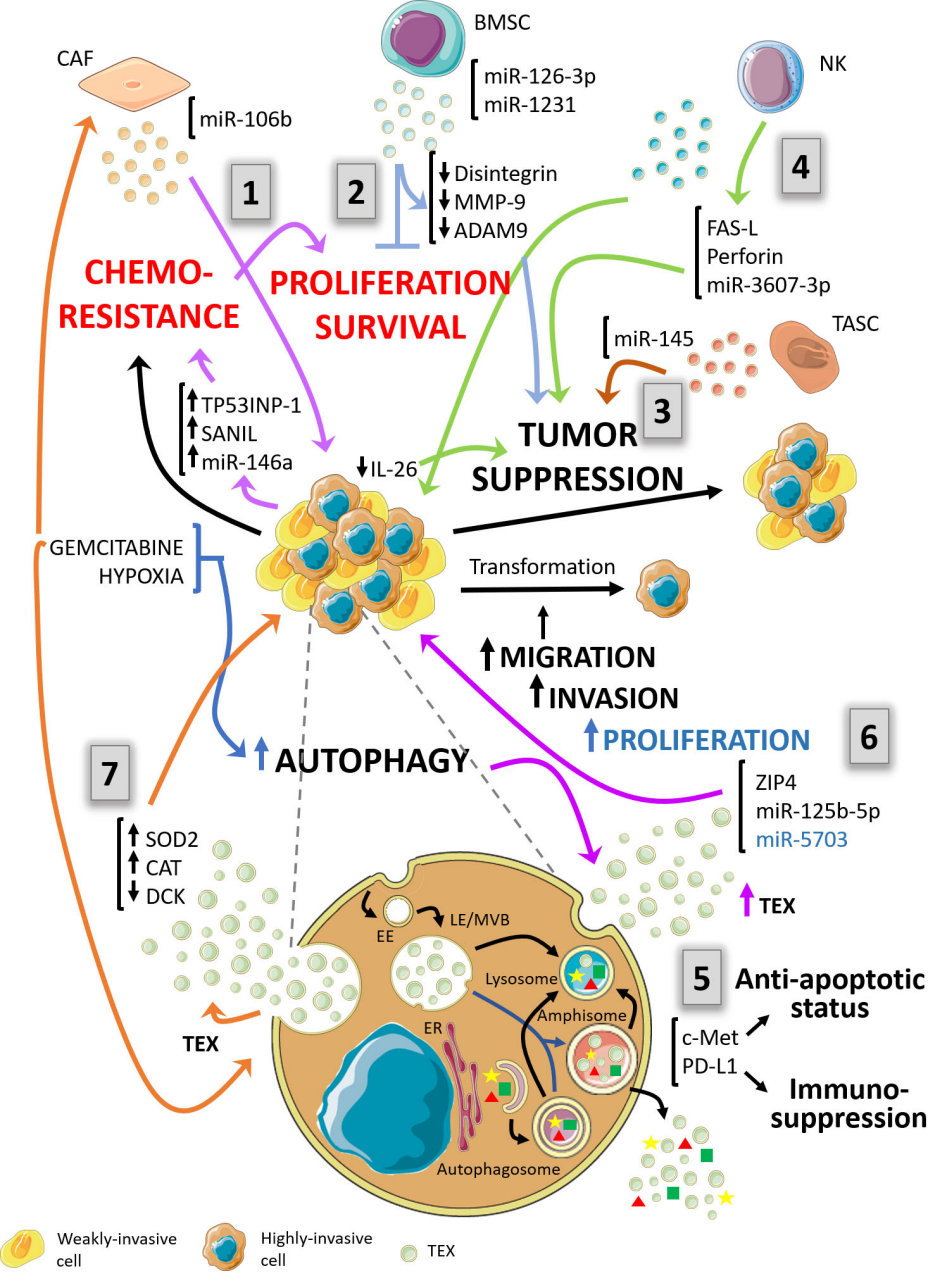
Schematic diagram depicting a proposal model of the complex relationship between autophagy and exosomes in the context of pancreas ductal adenocarcinoma (PDAC) and its environment. The autophagy pathway and exosomes biogenesis are suggested to be highly interconnected. Both pathways share several structures of the endo-lysosomal system. In the context of PDAC and its tumor microenvironment, autophagy and exosomal processes are mutually influenced and condition tumor behavior responding to the pressure of the immune system. (1) – *violet arrows* - Cancer-associated fibroblasts (CAFs) foster proliferation and chemoresistance. Exosomes bearing miR-106b are released by gemcitabine-treated CAFs increasing proliferation and survival of PDAC cell lines. The effector response is traduced in cancer cells as induction of chemoresistance-inducing factors, Snail, and miR-146a, and TP53INP1. (2) – *light blue arrows* - Bone marrow mesenchymal stem cell (BMSC)-derived exosomes contain miR-126-3p and miR-1231, which in turn inhibit proliferation, invasion and metastasis and promotes apoptosis by down-regulation of disintegrin and metalloproteinase-9 (ADAM9). (3) – *brown arrows* - Vesicles from tumor-associated stroma cells (TASC) are enriched in miR-145. This molecule possesses tumor suppressor action on target cells by inducing apoptosis. (4) – *green arrows* - NK-derived exosomes mediate tumor cells suppression by two mechanisms. One of them is Fas-FasL interaction between exosomes and tumoral cells. The other mechanism is mediated by excessive uptake by cancer cells of exosomes carrying miR-3607-3p which possess tumor suppressive qualities and decrease IL-26 expression. (5) – *purple arrows* – PDAC tumor exosomes (TEXs) bearing c-Met and PD-L1 enhance carcinogenesis. c-Met provides proliferation, migration, and an anti-apoptotic status in recipient cancer cells. PD-L1 guarantees evasion of immune control. (6) – *black arrow* – TEXs from highly invasive cells carry ZIP4, miR-125b-5p and miR-5703 towards weakly invasive cancer cells enhancing the aggressiveness of these last and promoting an increased invasive potential. (7) – *orange arrows* – In response to gemcitabine treatment, chemoresistant PDAC cancer cells are capable of transferring their resistance properties to neighboring cells through exosomes. They release TEXs bearing miR-155 and induce upregulation of SOD2 and CAT meanwhile DCK, a gene related to gemcitabine metabolism, is downregulated. Furthermore, exosomes from different cell types of tumor microenvironment condition autophagy response and affect PDAC behavior. EE, Early endosomes; LE/MVB, Late Endosomes/Multivesicular bodies; ER, endoplasmic reticulum; TEX, Tumor-derived exosomes.

Available data suggest that exosomes, EVs in general, are changing the communication paradigm within the TME (**Figure 1**). These tiny vesicles can modulate both the immune and therapeutic responses in complex and difficult-to-treat pathologies such as the PDAC. The scientific community is just beginning to understand the mechanisms that govern the intricate and complex interactions among the different actors into the TME. In this scenario, autophagy seems to play a key role in exosomal biogenesis regulation and probably also in cargo selection. We still have a long way to go but is for sure that a future with an exciting new comprehension about tumor biology is waiting.

## Author Contributions

This review was drafted by DP, MG, DG, and EA, designed by DG and critically revised by DG and EA. Figure was elaborated by DP and MG. All authors contributed to the article and approved the submitted version.

## Funding

University of Buenos Aires 20020170100014BA.

## Conflict of Interest

The authors declare that the research was conducted in the absence of any commercial or financial relationships that could be construed as a potential conflict of interest.
